# Sister Joseph's nodule in a liver transplant recipient: *Case report and mini-review of literature*

**DOI:** 10.1186/1477-7819-3-4

**Published:** 2005-01-14

**Authors:** Fabrizio Panaro, Enzo Andorno, Stefano Di Domenico, Nicola Morelli, Giuliano Bottino, Rosalia Mondello, Marco Miggino, Tomasz M Jarzembowski, Ferruccio Ravazzoni, Marco Casaccia, Umberto Valente

**Affiliations:** 1 Department of Transplant Surgery, St. Martino Hospital-University of Genoa, Genoa, Italy; 2 University of Illinois at Chicago, Department of Surgery, Division of Transplantation, Chicago, IL. USA

## Abstract

**Background:**

Umbilical metastasis is one of the main characteristic signs of extensive neoplastic disease and is universally referred to as Sister Mary Joseph's nodule.

**Case presentation:**

A 59-years-old Caucasian female underwent liver transplant for end stage liver disease due to hepatitis C with whole graft from cadaveric donor in 2003. After transplantation the patient developed multiple subcutaneous nodules in the umbilical region and bilateral inguinal lymphadenopathy. The excision biopsy of the umbilical mass showed the features of a poorly differentiated papillary serous cystadenocarcinoma. Computed tomographic scan and transvaginal ultrasonography were unable to demonstrate any primary lesion. Chemotherapy was start and the dosage of the immunosuppressive drugs was reduced. To date the patient is doing well and liver function is normal.

**Conclusions:**

The umbilical metastasis can arise from many sites. In some cases, primary tumor may be not identified; nonetheless chemotherapy must be administrated based on patient's history, anatomical and histological findings.

## Background

Metastases to the umbilicus are universally referred to as Sister Joseph's (or Sister Mary Joseph's) nodule. Etiology is related to the presence of primary malignant disease in the abdominal cavity or occasionally in the chest and/or breast [1-5]. Historically, Sister Mary Joseph (1856–1939) was a surgical assistant under the guidance of Dr. William Mayo. She was the first one to note the connection between the umbilical nodule and intra-abdominal cancer. The first case reporting the presence of Sister Mary Joseph's nodule was in 1864 by Storer, however; Hamilton Bailey was the first one to use the term "Sister Mary Joseph's nodule.

Although skin metastasis is rare and range between 5% and 9%, it is estimated that 1% to 3% of abdomino-pelvic tumors metastasize to the umbilicus [2,3,5]. The most common primary neoplasm is adenocarcinoma (75%), more rarely squamous cell carcinoma followed by undifferentiated tumors or carcinoid can metastasize to umbilicus. In men, gastrointestinal tract (55%) is the most common location of the primary neoplasm that metastasizes to the umbilicus, followed by stomach, colon, rectum, small bowel, and pancreas in a decreasing order [1-5]. In women, on the other hand, gynecological neoplasms particularly ovarian cancer is the most common primary site, of which serous papillary cystadenocarcinoma (34%) is the most frequent. Further, endometrial carcinoma, cervix, vagina and vulva may also be responsible for the metastasis to the umbilicus. In addition to these the literature reports other but rare sites that could form potential grounds for metastasis to the umbilicus, namely gallbladder, liver, breast, lung, prostate, penis, peritoneum, lymphoma, bladder and kidney. In about 11% of the umbilical metastasis, the origin of the metastasis is unknown [4,5]. Upon recognition of the positive nodules in the umbilicus, physician must consider other potential sources such as endometriosis, melanocytic nevi, fibroma, epithelial inclusion cysts, seborrheic keratosis, pilonidal sinus, keloid, foreign body granulomas, myxoma, omphalitis, abscesses, umbilical hernia and of course primary malignant umbilical tumor (melanoma, squamous and basal cell carcinoma, sarcoma and adenocarcinoma) [6]. In many instances, Sister Joseph's nodule is the first and often the only indication of an underlying or occult cancer and/or may indicate recurrence of previously treated malignancy. The presence of umbilical metastasis usually suggests already advanced metastatic process characterized by poor prognosis [4,5]. Therefore, a biopsy of umbilical nodule is recommended which is safe and easy. Obtaining histological description can further direct the clinician to dictate a prompt treatment. In the case of recurrence of the neoplasm, fine needle aspiration cytology may be sufficient to provide definite diagnosis; however, sometimes clinical, cytological, histological, radiological and/or surgical investigations may not be sufficient to identify primary site of the metastasis [7].

Serous papillary cystadenocarcinoma has been documented previously as a cancer responsible for Sister Joseph's nodule because of the metastasis to the regional lymph nodes [6-9]. Herein, we report, to the best of our knowledge, the first case of Sister Joseph's nodule in a patient after liver transplantation complicated by serous papillary cystadenocarcinoma. In addition, we review the potential routes by which tumor spread may have occurred and the prognostic significance associated with umbilicus metastasis.

## Case presentation

A 59-year-old Caucasian woman was admitted to our hospital for progressively enlarging but painless mass in the region of the umbilicus. The umbilical mass of approximately 5 cm in diameter and has developed two months after liver transplantation. The nodule was firm with irregular borders, wine-red shade and fixed to the surrounding tissues. The overlying skin was not ulcerated. She never smoked, consumed alcohol or used oral contraceptive pills. The gynecological history was unremarkable. The patient underwent a liver transplantation for end stage liver disease (ESLD) secondary to hepatitis C in mid of 2003. Prior to the transplant, her serum alpha-fetoprotein (alpha-FP) and CEA concentration were measured and both were within normal limits. The transplant procedure and immediate post-transplant period were unremarkable. The post-transplant immunosuppressive regimen consisted of Tacrolimus (6 mg/day) and steroids (prednisone 10 mg/day). Two months after transplantation, during routine follow-up, physical examination revealed multiple subcutaneous satellite nodules in close proximity to the umbilical region and lower abdominal wall. There was bilateral inguinal lymphadenopathy. A plain anterior/posterior chest x-ray was normal. Ultrasound of the abdominal cavity showed a 4 × 4 cm mass below the umbilicus and additional smaller nodules confined to the lower abdominal wall. Paraaortic lymph nodes were not identified. The echogenicity of the mass consisted of alternative hyper- and hypoechoic with poorly defined edges. However, the general condition of the patient was good and the laboratory test showed: hemoglobin level 9.6 g/dl, total bilirubin 0.6 mg/dl, conjugated bilirubin 0.1 mg/dl, Alkaline Phosphates 273 U/l, GGT 40 U/L, AST 36 U/L, and ALT 56 U/L. There was serological evidence of past hepatitis C virus infection. The biopsy of the umbilical mass showed features characteristic for a poorly differentiated serous papillary cystadenocarcinoma. An extended resection of the umbilical and paraumbilical metastasis was performed. In addition, extensive investigation to detect the primary neoplastic origin was initiated. Tumor marker evaluation consisted of: CEA 0.22 [normal 0–3.7 ng/ml], alpha feto protein 1.49 (normal: 0.8–9.4 UI/ml), CA125 27.73 (normal: 0–30 UI/ml), CA 19.9 was 8.61 (normal: 0–36.2 UI/ml), CA 15.3 was 22.48 (normal: 0–35 UI/ml). Multiple tumor marker measurements were obtained and each time similar results were reported. A computed tomographic (CT) scan was unable to demonstrate any lesion that could be considered a primary source of the metastasis to the umbilicus. Trans-vaginal ultrasonography showed a normal uterus and ovaries; ultrasonographic examination of thyroid and breast did not show any pathological changes as well. In spite of laborious efforts, the tumor's primary site was not found at that time. However, considering the histological results, the umbilicus metastasis could be attributed almost entirely to the ovarian cancer stage 4, since the main primary site in females are the ovaries. In order to prevent further tumor extension and expansion, tacrolimus was reduced from 6 mg/day to 1.5 mg/day. In addition, chemotherapy with Taxol was initiated (50 mg/m^2 ^I.V) once a week for 16 weeks. To date, after 6 months of follow-up, the patient is doing well, the liver function is normal and tacrolimus serum level is 1 ng/ml. A recent whole body CT-scan showed regression of iliac and inguinal lymph nodes involvement.

## Discussion

Metastases may appear virtually anywhere in the body; however, certain sites are more common then others and umbilical metastasis are unusual sites [9,10]. In majority of cases, the metastatic lesions accompany the symptoms and signs of the primary tumor; however, it can be the first and often the only sign of a carcinoma, although this happens rarely [10]. A variation in vascularity and embryological development makes the umbilicus easy target for metastasis from an intra-abdominal tumor. In fact, during fetal development, the ductus venous (ductus venous Arantii) connects the umbilical portion of the left branch of the portal vein to the inferior vena cava, thus shunting oxygenated umbilical cord blood away form the liver. After birth the duct obliterates and persists as ligamentum venosum or Arantius' ligament. Because of its functional role, it is commonly believed that the ligament runs from the left branch of the portal vein to the vena cava itself. However, attention to anatomical detail demonstrates that the fibers of the ligament insert either on the left hepatic vein or at the junction between the left hepatic and the middle hepatic vein [1,11]. We know that in cirrhotic patients, the umbilical vein remains open due to portal hypertension.

There are numerous potential routes by which a carcinoma can metastasize to the umbilicus in a liver transplant recipient. Malignant carcinomatous cells in the portal venous system may reach the umbilicus by way of a patent umbilical vein [12-15]. This would be more likely to occur in the presence of portal hypertension and the resulting portal-systemic shunting of blood [16]. Since our patient received liver transplant for cirrhosis secondary to hepatitis C virus infection accompanied by portal hypertension, this could be a possible mode of spread of papillary serous cystadenocarcinoma in our patient. In addition, presence of severe esophageal varicies and large umbilical vein that was viewed by angiography examination before the liver transplantation could have contributed to the spread of tumor. Therefore, in this case, malignant cells could have reached the umbilicus via the umbilical vein. In addition, a pelvic carcinoma of unknown origin may spread via lymph nodes to the umbilical region [9,17,18]. Furthermore, dermal lymphatics are a potential route of spread to the umbilicus and should be considered, since the umbilicus can be reached by retrograde lymphatic flow [4]. Nevertheless, the spread of malignant cells could have occurred via embolization thru the arterial blood supply to the umbilicus. Furthermore, direct implantation of tumor cells may have occurred via direct extension of the neoplasm from the anterior peritoneal surface or via redistribution of the peritoneal fluid flow [19,20]. Finally, cutaneous metastasis to the anterior abdominal wall, may perhaps cause malignant cells to spread in a retrograde direction, namely from the metastatic lesions along the subcutaneous lymphatic vessels to the umbilicus. It is known that the immunosuppression promotes tumor spreading and potentates its expansion, thus immunosuppressive regimen plays a crucial role in the tumor expansion and metastasis.

Umbilical metastasis is one of many characteristic signs of extensive neoplastic disease. It suggests advanced distant metastasis and is associated with poor prognosis; mean survival is approximately 10–12 months, although long-term survival has been reported, but only in the presence of solitary metastatic umbilicus nodule [5].

## Conclusions

Presence of umbilical nodule or nodules requires extensive, meticulous and laborious evaluations since the differential diagnosis for umbilical nodule are many. In our case although, the primary tumor was not found, we treated it as ovarian tumor considering the histological findings and the iliac-inguinal nodes involvement.

## Competing interests

The author(s) declare that they have no competing interests.

## Authors' contributions

**FP**: conception and design, interpretation of data, drafting the article and revising

**EA**: interpretation of data, drafting the article and revising

**SDD**: acquisition of data and revising

**NM**: interpretation of data, design and coordination and helped to draft the manuscript

**GB**: collection of data, design and coordination and helped to draft the manuscript

**RM**: collection of data, design and coordination

**MM**: acquisition of data and drafting the article

**TJ**: drafting the article and interpretation

**FR**: acquisition of data and design

**MC**: interpretation of data, drafting the article

**UV**: drafting the article, revising and supervision

**Figure 1:**
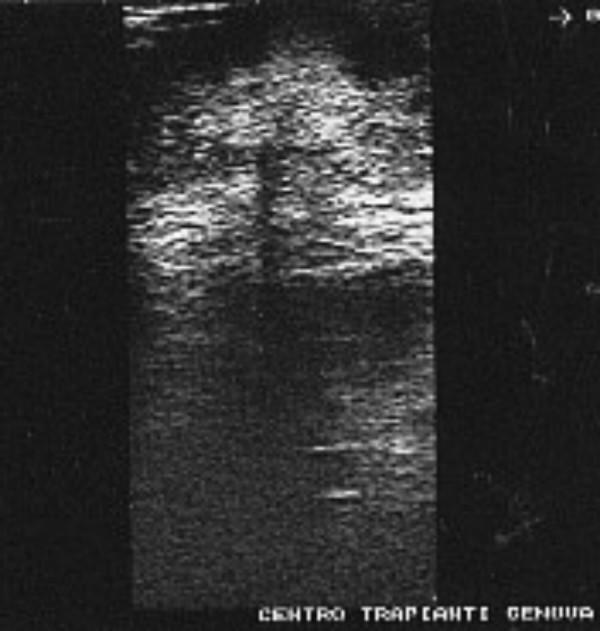
Preoperative Sister Mary Joseph's nodule ultrasonography: 4 × 4 cm mass confined below the umbilicus (arrows). The main lesion is partly hyperechoic and partly hypoechoic with a poorly defined edge.

**Figure 2:**
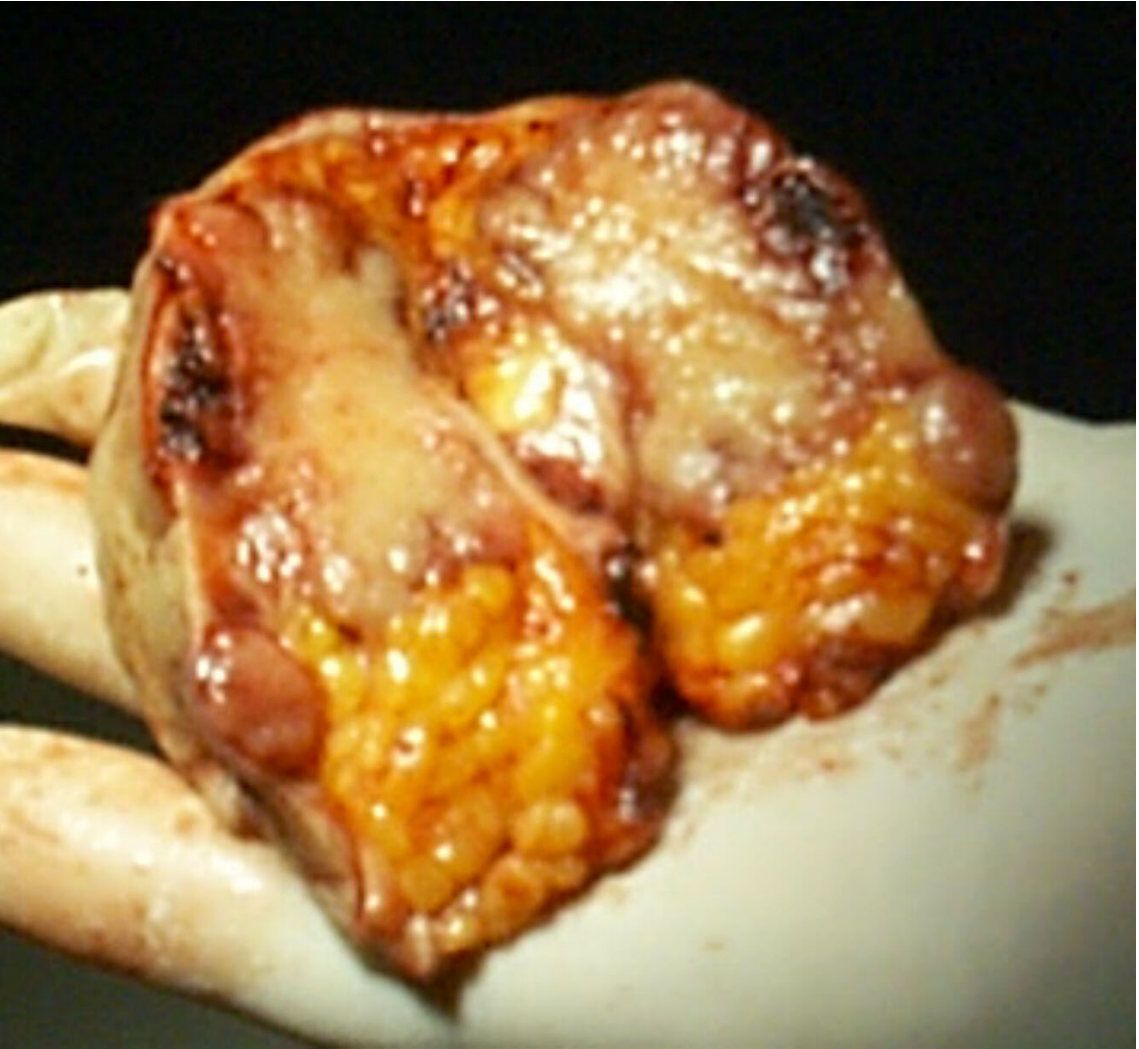
Intraoperative specimen of the umbilical region: Sister Mary Joseph's nodule and umbilicus.
